# A randomized trial of practice facilitation to improve the delivery of chronic illness care in primary care: initial and sustained effects

**DOI:** 10.1186/1748-5908-8-93

**Published:** 2013-08-22

**Authors:** Michael L Parchman, Polly H Noel, Steven D Culler, Holly J Lanham, Luci K Leykum, Raquel L Romero, Raymond F Palmer

**Affiliations:** 1 MacColl Center for Healthcare Innovation, Group Health Research Institute, Group Health Cooperative, 1730 Minor Ave Suite 1600, Seattle, WA, USA; 2 VERDICT Health Services Research Program, South Texas Veterans Health Care System, San Antonio, TX, USA; 3 Department of Medicine, University of Texas Health Sciences Center San Antonio, San Antonio, TX, USA; 4 Rollins School of Public Health, Emory University, Atlanta, GA, USA; 5 McCombs School of Business, University of Texas, Austin, TX, USA; 6 Department of Family and Community Medicine, University of Texas Health Sciences Center-San Antonio, San Antonio, TX, USA

## Abstract

**Background:**

Practice facilitation (PF) is an implementation strategy now commonly used in primary care settings for improvement initiatives. PF occurs when a trained external facilitator engages and supports the practice in its change efforts. The purpose of this group-randomized trial is to assess PF as an intervention to improve the delivery of chronic illness care in primary care.

**Methods:**

A randomized trial of 40 small primary care practices who were randomized to an initial or a delayed intervention (control) group. Trained practice facilitators worked with each practice for one year to implement tailored changes to improve delivery of diabetes care within the Chronic Care Model framework. The Assessment of Chronic Illness Care (ACIC) survey was administered at baseline and at one-year intervals to clinicians and staff in both groups of practices. Repeated-measures analyses of variance were used to assess the main effects (mean differences between groups) and the within-group change over time.

**Results:**

There was significant improvement in ACIC scores (p < 0.05) within initial intervention practices, from 5.58 (SD 1.89) to 6.33 (SD 1.50), compared to the delayed intervention (control) practices where there was a small decline, from 5.56 (SD 1.54) to 5.27 (SD 1.62). The increase in ACIC scores was sustained one year after withdrawal of the PF intervention in the initial intervention group, from 6.33 (SD 1.50) to 6.60 (SD 1.94), and improved in the delayed intervention (control) practices during their one year of PF intervention, from 5.27 (SD 1.62) to 5.99 (SD 1.75).

**Conclusions:**

Practice facilitation resulted in a significant and sustained improvement in delivery of care consistent with the CCM as reported by those involved in direct patient care in small primary care practices. The impact of the observed change on clinical outcomes remains uncertain.

**Trial registration:**

This protocol followed the CONSORT guidelines and is registered per ICMJE guidelines: Clinical Trial Registration Number: NCT00482768.

## Background

Practice facilitation (PF) is an especially promising approach to supporting primary care redesign [1-3]. PF occurs when a trained facilitator provides support services to a primary care practice for an improvement initiative. The PF approach enables teams to overcome challenges encountered when implementing changes in the office setting by building their internal capacity to engage in redesign or improvement efforts [4]. Facilitators assist teams within the practice as they identify and prioritize areas of change as well as help them develop tailored action plans for improvement. PF has also been referred to as quality improvement coaching or practice enhancement assistance [2]. A recent systematic review suggests that PF is a robust intervention for improving the adoption of evidence-based preventive care guidelines in primary care [1].

The Chronic Care Model (CCM) describes elements of an approach to providing chronic care in ambulatory care settings with proven effectiveness [5-7]. Studies of CCM implementation and other primary care redesign efforts suggest that practice change/redesign is difficult [7]. Change can be especially challenging in small, autonomous primary care practices where resources are scarce and where the environment external to the practice is not supportive of change [8]. PF is emerging as a promising approach for assisting practices in overcome these challenges. Although effective at improving guideline adoption for preventive care, such as colon cancer screening [1], the ability of PF to change how the primary care team organizes itself around chronic illness care remains unknown.

Here we report the results of group-randomized trial of a PF intervention to improve the organization and delivery of diabetes care in small, autonomous primary care practices. The specific objective is to examine changes in the degree to which care is organized around the CCM at the conclusion of the one-year intervention, its sustainability one year after withdrawal of the PF intervention, and subsequent improvement in the delayed intervention (control) practices.

## Methods

This was a group-randomized trial launched in the fall of 2007 in the South Texas region of the United States. The study design of this trial and details about the intervention have been previously reported [9]. Briefly, the study was conducted in small, autonomous primary care clinics or ‘practices’ in South Texas. These urban, suburban, and rural practices, each with one to three clinicians, serve a population of primary care patients diverse in demographic characteristics, insurance coverage, and healthcare needs. Subjects for this study were the clinicians and staff at participating practices.

### Recruitment

Due to travel distance and costs, recruitment was limited to the San Antonio Metropolitan Statistical Area and surrounding counties within a one-hour drive of the medical center. Early recruits included 10 active members of a primary care Practice-Based Research Network (PBRN), all of whom agreed to participate. PBRN enrollees were asked to recommend colleagues whom they thought might be interested in study participation who also referred colleagues (n = 25). These physicians were contacted directly by phone and in-person recruiting visits were scheduled at their offices resulting in 22 participants. In addition, 145 recruitment letters were sent to primary care physicians within the region identified from professional society membership guides. From these letters, 15 practices responded; of those, eight agreed to participate in the study, resulting in a total of 40 practices. The sample size of 40 practices was determined from measurement of the primary outcome, the CCM score, across 20 clinics resulting in a power of 0.94 to detect a change in the CCM score of at least 1.5 in response to the intervention as described in the original published protocal [9].

### Randomization

A stepped-wedge study design was used with block randomization of practices in groups of 10 to either an ‘initial intervention’ or ‘delayed intervention’ arm of the study [10]. The random allocation sequence was generated by a member of the study team (RP) after each group of 10 clinics was recruited. A stepped-wedge design is a type of crossover design in which different clusters cross over (switch treatments) at different time points. For this study, 20 practices, the ‘initial’ intervention practices, were randomized to receive the PF intervention for one year,’ while the remaining 20 practices served as controls. Following the completion of the one-year intervention and withdrawal of facilitators from the initial intervention practices, the ‘control’ practices were crossed-over to receive a one-year ‘delayed intervention’ of PF. Randomization was done before the orientation visit where consent for study participation occurred because status of randomization was needed to inform length of and type of data collected during the initial visit. For example, interviews with clinicians and staff were conducted during and after the team meeting introducing the study in practices randomized to the initial intervention arm but not the delayed intervention.

### Intervention

Practice Facilitators held a minimum of six one-hour team meetings within each practice over a 12-month period of time. As is common with many previous PF efforts, baseline chart audit and feedback as well as interactive consensus building and goal setting were incorporated into the intervention [1]. For the audit and feedback component, glycosylated hemoglobin (HbA1c), blood pressures, and lipid levels were audited from the medical records of a random sample of 30 patients seen in the prior 12 months with a diagnosis of type 2 diabetes. Additionally, 60 consecutive adults presenting for care in each practice completed a satisfaction survey. The PF intervention in each practice began with a review of the chart audit results, as well as results from the patient survey.

Each Practice Facilitator was trained in the use of multiple ‘tools’ within a ‘toolbox’ to assist practices with improving their chronic illness care. These tools included: group/shared medical appointments; a diabetes registry; point-of-care HbA1c testing; resources/approaches to patient education/activation; and planned diabetes visits with clinical reminders and decision support for providers and staff. After presenting results from the chart audit and patient survey, along with the five strategies in the toolkit, the facilitators worked with clinicians and staff to identify alternative approaches or to adapt strategies from the toolbox to improve the delivery of diabetes care through a process of interactive consensus building and goal setting in each practice.

### Data collection and measurements

Data were collected at three points in time, approximately one year apart in each practice. Wave one or ‘baseline’ data were collected when facilitators made an initial visit to each participating initial intervention and delayed intervention practice to explain the study, obtain consent from participants, and administer a baseline survey to clinicians and staff. Following Wave one data, Wave two data was collected one year after the initiation of the PF intervention in the practices randomized to the initial intervention, and at the start of the PF intervention for those randomized to the delayed intervention (control) practices. Wave three surveys were collected one year after the completion of the intervention in the initial intervention practices, and one year after the beginning of the PF intervention in the delayed intervention (control) practices.

The clinician/staff survey included the Assessment of Chronic Illness Care (ACIC) survey to measure the extent to which the care delivered in each practice was consistent with the elements of the CCM, the ‘CCM score’ [11]. Each item is scored on a 0 to 11 scale and provides sub-scale scores for each of the six CCM components as well as a total score. Scores from 0 to 2 represent ‘limited support,’ 3 to 5 represent ‘basic support,’ 6 to 8 is ‘good support,’ and 9 to 11 represent ‘fully developed support’. Version 3.5 of the ACIC was used in this study; in addition to the six subscales, it also includes items that address how well a practice integrates the CCM elements.

### Analysis

After surveys were scanned into a database, the data were examined for outliers and missing values. Missing data in the surveys were addressed using multiple imputation following the recommended procedures [12,13]. The CCM scores from the ACIC survey were calculated as recommended by the originators of the ACIC instrument [11]. Prior work suggests that survey measures of chronic illness care through surveys of practice members may vary by role within the practice and are more valid if measured by clinicians and those involved in direct patient care compared to other office staff [14,15]. Cognizant of these findings, we limited our analysis of the ACIC surveys to those returned by those in direct patient care (physicians, advanced nurse practitioners and physician assistants, RNs, and LVN or LPNs) in each practice. Delivery of care consistent with the CCM is a practice-level construct, therefore a mean ACIC score for each practice was calculated for each of the three waves of data collection. We used a full factorial repeated-measures analysis of variance (ANOVA) to assess the main effects (mean differences between groups) and the within-group change over time. This approach allowed us to determine whether one group changed more rapidly over time (group-by-time interaction).

## Results

A total of 40 practices were recruited for participation in the study and randomized. The enrollment visit for the first practice occurred in October of 2007, the final site visit in the study occurred in December of 2012. The trial was not completed until all practice sites had 12 months of the practice facilitation intervention. Nineteen of the 20 practices completed the initial intervention. In one practice assigned to the initial intervention, delays in initiating the PF intervention due to practice relocation, implementation of an electronic medical record, and staff turnover delayed the PF intervention by almost two years. As a result, they were reassigned to the delayed intervention group. Two practices assigned to the delayed intervention failed to complete both baseline and one-year follow-up assessments and are not included in this analysis. A total of 38 practices, 19 practices in each arm of the study, had complete data for analysis. Characteristics of practices and their patient populations are shown in Table 1.

**Table 1:** Characteristics of practice members and practices

**Practice member characteristics (n = 280 baseline surveys)**	
Profession (%)	
MD or DO	15.4
PA	2.9
NP	3.6
RN/LVN	5.4
Medical Assistant	31.8
Receptionist	12.1
Office Manager	7.5
Other	21.4
Female (%)	82.5
Level of Education (%)	
High School	20.8
Vocational or Some College	44.2
College Degree or Higher	35.0
Age in Years, Mean (SD)	37.3 (11.8)
Years Worked at Practice, Mean (SD)	4.6 (5.9)
Practice characteristics (n = 38)	
Number of providers (MD,DO,PA,NP), Mean (SD), Range	1.55 (0.9) 0-4
Mean (SD) Number of non-provider staff, Mean (SD), Range	5.5 (4.2) 2-26
Office Visits per Day per FTE, Mean (SD), Range	23.0 (5.5) 12.5-37.5
Percent of Medicaid patients, Mean (SD), Range	12.3 (16.2) 0-80
Percent of Medicare patients, Mean (SD), Range	32.8 (21.5) 0-80
Percent of Non-Hispanic White patients, Mean (SD), Range	28.9 (18.7) 0-60
Practices with Computerized Health Record (%)	51.3

Clinicians and nurses returned 77 baseline surveys, 80 surveys at wave two, and 77 surveys at wave three. The overall response rate at each wave was 98%. Practices in the initial intervention group received a mean of 6.7 (SD 1.2, Range 5–10) facilitation visits between wave one and wave two and those in the delayed intervention (control) group received 7.2 visits between wave two and wave three (SD 1.5, Range 5–10), (p = 0.33). On average, each facilitator worked with eight to ten practices at one time. Table 2 describes strategies chosen by the practices in response to the facilitation intervention across the initial intervention group. Although practices chose to work on several components of the CCM in response to the feedback report and team meetings with the facilitator, most practices chose to work on patient self-management support. All practices worked on more than one change. In addition to strategies that focused on improving diabetes care, many practices initiated regular team meetings for the first time.

**Table 2:** Practice facilitation strategies chosen by the initial intervention group (n = 19)

**CCM element addressed**	**Number of practices**	**Description of a common intervention activity**
Self management support	16	Provided patients with logbooks to track their HbA1c, BP and Lipid levels; patient education binders in exam rooms; diabetes education videos; ‘Ask me about Diabetes ABCs’ buttons worn by staff; diabetes posters in exam rooms.
Delivery system design	8	Group visits; re-organized clinic staff and delegated authority for testing/immunizations, etc.; began point-of-care HbA1c testing;
Clinical information systems	5	Improved EMR functionality to provide individual and population level reports; diabetes flow sheets and/or templates in charts;
Community linkages	2	Developed linkages for referral to nutritionist or for eye exams.
Prepared proactive teams		
Initiated or increased staff meetings	8	Many practices had never met to discuss clinical improvement activities or at least met infrequently. Practice facilitators modeled effective meeting techniques and taught practices how to hold productive, effective team meetings.
Initiated Huddles	9	Huddles are quick 3–5 minute meetings of practice staff during the day for the purpose of planning for changes in workflow, anticipating and solving problems, and making adjustments to insure everyone is ‘on the same page’.

At baseline, most practices fell into the ‘basic’ or ‘good support’ for chronic illness care with an overall mean ACIC score across all practice sites of 5.67 (SD 1.86) (Table 3). The range of mean practice-level scores was 2.15 to 9.65. There was no significant difference in mean ACIC scores at baseline for initial intervention practices, 5.59 (SD 1.89), and delayed intervention or control practices, 5.56 (SD 1.54).

**Table 3:** ACIC Scores at three waves

	**Wave 1: baseline**	**Wave 2**	**Wave 3**
**Initial intervention**			
ACIC score	5.59 (1.89)	6.33 (1.50)*	6.60 (1.94)
ACIC category (#clinics)			
Basic support	n = 9 (47.4%)	n = 3 (15.8%)	n = 5 (26.3%)
Good support	n = 9 (47.4%)	n = 14 (73.7%)	n = 11 (57.9%)
Fully supported	n = 1 (5.3%)	n = 2 (10.5%)	n = 3 (15.8%)
**Delayed intervention (Controls)**			
ACIC score	5.56 (1.54)	5.27 (1.62)	5.99 (1.76)
ACIC category			
Basic support	N = 7 (36.8%)	N = 9 (47.4%)	N = 5 (27.8%)
Good support	N = 10 (52.6%)	N = 9 (77.4%)	N = 11 (57.9%)
Fully supported	N = 2 (10.5%)	N = 1 (5.3%)	N = 3 (16.7%)

*Wave 1 to Wave 2 paired t-test = −2.38 (p = .02), mean difference 0.75 (S.D.1.36), 95% CI of the difference 0.09, 1.40.

**Figure 1:**
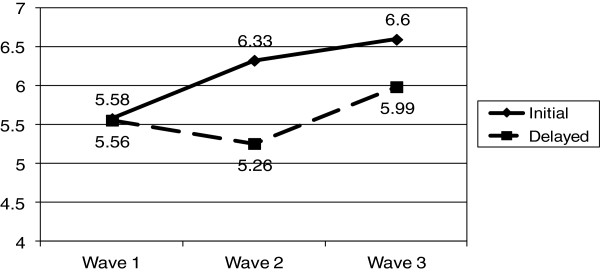
ACIC scores: initial and delayed intervention practices.

ACIC scores for intervention and control practices for all three waves of data collection are shown in Table 3 and the Figure 1. The repeated measures ANOVA revealed a significant within-subjects effect accounting for a significant increase in ACIC over time. The between subjects (*e.g.*, group) effect was not significant, however the time by intervention interaction term was significant, (F = 4.26, p = 0.046), indicating a significant difference in ACIC scores between the initial intervention and delayed intervention groups. Table 3 also provides the number and proportion of clinics in each ACIC category (limited, basic, good, full) across the three waves of data collection. Within the initial intervention group, 10 of the 19 clinics were in the ‘good’ or ‘fully’ supported ACIC category at baseline and 16 of the 19 clinics were in these categories after the PF intervention (wave two data). A similar pattern can be seen for the delayed (control) intervention clinics from wave two to wave three, after the PF intervention.

A visual inspection of the ACIC means shown in the Figure 1 suggests that the significant time by intervention term may have occurred from wave one (baseline) to wave two. This was confirmed by a paired t-test that showed a significant difference in ACIC scores for the initial intervention group from baseline to wave two. The mean difference in CCM score from wave one to wave two for the intervention practices was 0.75 (95% CI of the difference 0.09, 1.40; p = 0.02), mean difference. Although the proportional improvement in CCM score (13.9%) in the delayed group from wave two to wave three (before and after the PF intervention) was almost equal to the change in the initial intervention group between wave one and wave two (14.4%), the improvement did not reach statistical significance. Of note is the sustained and slight increase in ACIC score in the initial intervention group one year after withdrawal of the PF intervention.

## Discussion

There was significant improvement in the degree to which care was consistent with the CCM in small primary care practices randomized to the PF intervention compared to those in the delayed intervention (control group). This improvement was sustained one year after completion of the PF intervention. These findings are consistent with prior studies which demonstrated the effectiveness of PF [1]. An analysis of sub-scores from the ACIC revealed the biggest change occurred in the area of self-management support score, which increased from 4.39 to 5.83 among practices in the initial intervention group, compared to a slight decline from 5.22 to 4.95 in the delayed intervention (control) group. Such a sub-score change is consistent with the finding that 16 of the 19 intervention practices made improvement efforts within this area as shown in Table 2.

Why should PF result in such sustained improvement? When an organization and its members encounter a change intervention, such as redesigning the way they deliver chronic illness care, they must often shift goals and learn to solve non-routine problems, often under time pressure and turbulent conditions. Primary care practices and the diverse individuals in them must continually make sense of new environmental cues as a team and develop a collective meaning of new or stressful situations if they are to successfully and consistently implement change [16]. Research across different healthcare settings has demonstrated the importance of improving relationships and sense-making activities within teams when designing interventions [17-20]. It is possible that the PF intervention was effective because the facilitation improved relationships among the practice members and enabled conversations within teams during their team meetings that allowed them to collectively make sense of the changes they were asked to make [19].

It is also important to note that the ACIC scores were sustained one year after withdrawal of the PF intervention, and in fact, improved slightly. This suggests that the PF intervention may have changed how information is shared and used in the practice and how practice members accomplished tasks that proved enduring over time [21]. That is, PF changed practice member relationships in ways that improved the ability of the practice team to make sense and learn from the intervention, leading to sustainability [19].

The change in self-assessed chronic illness care, although was significant, was relatively small, from 5.59 at baseline to 6.60 one year after the PF intervention. This raises questions about its relevance for improved clinical outcomes. Prior work has demonstrated a relationship between ACIC score and control of HbA1c [22-24]. In one study, a one-point increase in the ACIC score was associated with a 0.144% decrease in HbA1c; thus, it is possible that the observed one-point improvement in ACIC scores seen one year after the PF intervention may result in improved clinical outcomes [22]. One explanation for the small observed change in ACIC scores is that development of a common understanding of the desired goals and required changes needed to deliver better chronic illness care requires a longer period of time than one year. In fact, ACIC scores continued to improve in the year after the completion of the PF intervention, although the rate of change was less than during the year of facilitation.

It is possible that larger improvements in chronic illness care within a primary care practice are possible with a more intense intervention than the six to seven facilitated team meetings over a one-year period delivered in this study. Although the original intent of the study design was to have monthly facilitation visits over the 12-month intervention period, competing demands within the practices such as EMR implementation and staff turnover, prevented this higher number of visits. In addition, one may need to combine facilitation with other components, such as a learning collaborative, repeated performance reporting, and educational outreach visits [25]. Finally, one would be remiss not to mention the importance of the lack of financial incentives for these practices to make substantive change in the manner in which they deliver chronic illness care. These practices are small businesses and as such must consider the possible ‘return on investment’ in any change effort they undertake.

Change efforts within some practices were stymied by other changes that occurred during the intervention. Staff turnover often created problems by delaying facilitation visits or suspended visits all together. For example, in one practice, the staff ‘champion’ for improving diabetes care left, and the practice ‘stalled’ in its efforts to make changes until several months later when another staff member took on this role near the end of the facilitation intervention. In addition to staff turnover, several practices experienced other significant changes during the one-year PF intervention such as moving the practice location (three practices in the initial intervention group and three practices in the delayed intervention group), and implementation of an electronic health record (three practices in the initial intervention group and four practices in the delayed intervention group).

This study is also limited by the geographic restriction to a narrow region of the United States, and potential for selection bias inherent the selection of practices utilized for the study. In addition, the participating primary care practices were small, and different results might have been obtained using larger primary care practices, especially those embedded within integrated healthcare systems. The potential for selection bias favoring practices that are eager to improve also exists. However, these practices also tend to be the ones that are higher performing at the start of the study, making it more difficult to demonstrate significant and sustained improvements.

The study also has a number of strengths, including a diverse sample of ‘real-world’ practices, the use of theory to inform the intervention design, the randomization of practices to intervention and delayed intervention (control) groups, and the low rate of attrition of practices from the study. The impact of the PF intervention is demonstrated by the finding that 16 out of 19 practices showed improvement on the self-managemetn sub-scale.

## Conclusions

Practice facilitation resulted in significant and sustained improvement in the degree to which chronic illness care was consistent with the CCM in small primary care practices, although the clinical impact of the degree of improvement on patient outcomes is uncertain. Given the current challenges and ongoing efforts to redesign primary care into Patient-Centered Medical Homes across the United States, many are calling for the development of an infrastructure that supports the use of practice facilitators or coaches should [26,27]. This study adds additional support for these initiatives as it adds to the growing body of evidence that practice facilitation is an effective intervention for sustained improvement in the how care is delivered in small primary care practices. Next steps include linking organizational and process changes that result from practice facilitation to improvements in clinical outcomes, patient experience, and cost-containment.

## Competing interests

The authors declare that they have no competing interests.

## Authors’ contributions

MP designed the study and led the study team through all phases of the research. PN participated in the implementation of the study protocol, assisted with development of survey instruments, analysis of the data, interpretation of results, and assisted with manuscript preparation. SC participated in designing the data collection methods and contributed to modification of the intervention, analysis and interpretation of results, and contributed to writing of the manuscript. HL and LL both participated in adapting the intervention to the study sites, interpretation of results and writing of the manuscript. RR led the intervention team, assisted with data collection and helped the team with interpretation of findings and writing the manuscript. RP participated in developing the analytic design of the study, assisted with data collection and management, conducted the primary analysis and helped with writing the manuscript. All authors read and approved the final manuscript.
